# The Canadian Breast Cancer Symposium 2023 Meeting Report

**DOI:** 10.3390/curroncol31040135

**Published:** 2024-03-29

**Authors:** Tulin Cil, Jean-François Boileau, Stephen Chia, MJ DeCoteau, Katarzyna J. Jerzak, Anne Koch, Nancy Nixon, May Lynn Quan, Amanda Roberts, Christine Brezden-Masley

**Affiliations:** 1 Princess Margaret Cancer Centre, Toronto, ON M5G 2M9, Canada; tulin.cil@uhn.ca (T.C.); anne.koch@uhn.ca (A.K.); 2 Jewish General Hospital, Montreal, QC H3T 1E2, Canada; jean-francois.boileau@mcgill.ca; 3 British Columbia Cancer Centre, University of British Columbia, Vancouver, BC V5Z 4E6, Canada; schia@bccancer.bc.ca; 4 Rethink Breast Cancer, Toronto, ON M4M 3G3, Canada; mj@rethinkbreastcancer.com; 5 Sunnybrook Odette Cancer Centre, University of Toronto, Toronto, ON M4N 3M5, Canada; katarzyna.jerzak@sunnybrook.ca (K.J.J.); amanda.roberts@sunnybrook.ca (A.R.); 6 Department of Surgery and Oncology, University of Calgary, Calgary, AB T2N 4Z6, Canada; nancy.a.nixon@ahs.ca (N.N.); maylynn.quan@ahs.ca (M.L.Q.); 7 Marvelle Koffler Breast Centre, Mount Sinai Hospital, Toronto, ON M5G 1X5, Canada

**Keywords:** breast cancer, patient voice, systemic therapy, new advances, CNS metastases, surgical oncology

## Abstract

On 15–16 June 2023, healthcare professionals and breast cancer patients and advocates from across Canada met in Toronto, Ontario, for the 2023 Canadian Breast Cancer Symposium (CBSC.). The CBSC. is a national, multidisciplinary event that occurs every 2 years with the goal of developing a personalized approach to the management of breast cancer in Canada. Experts provided state-of-the-art information to help optimally manage breast cancer patients, including etiology, prevention, diagnosis, experimental biology, and therapy of breast cancer and premalignant breast disease. The symposium also had the objectives of increasing communication and collaboration among breast cancer healthcare providers nationwide and providing a comprehensive and real-life review of the many facets of breast cancer. The sessions covered the patient voice, the top breast cancer papers from different disciplines in 2022, artificial intelligence in breast cancer, systemic therapy updates, the management of central nervous system metastases, multidisciplinary management of ductal carcinoma in situ, special populations, optimization-based individual prognostic factors, toxicity management of novel therapeutics, survivorship, and updates in surgical oncology. The key takeaways of these sessions have been summarized in this conference report.

## 1. Introduction

The Canadian Breast Cancer Symposium (CBSC.) is a national, multidisciplinary event with the goal of developing a personalized approach to the management of breast cancer. On 15–16 June 2023, oncologists, surgeons, pathologists, nurses, residents and fellows, and patients met at the Omni King Edward Hotel in Toronto, Ontario, for the 2023 conference. The conference chairs were Christine Brezden-Masley MD PhD FRCPC, a medical oncologist at Mount Sinai Hospital (Toronto, ON, Canada), and Tulin Cil MD MEd FRCSC FACS, a surgical oncologist at the University Health Network (Toronto, ON, Canada).

Through 11 plenary sessions and two concurrent sessions featuring small-group discussions, experts provided state-of-the-art information to help optimally manage breast cancer patients, including etiology, prevention, diagnosis, experimental biology, and therapy of breast cancer and premalignant breast disease, as well as survivorship (Figure S1). The symposium also had the objectives of increasing communication and collaboration among breast cancer physicians and providers nationwide, and provided a comprehensive and real-life review of the many facets of breast cancer, including the most important one—the patient voice.

The sessions covered the patient’s perspective, the top breast cancer papers from different disciplines in 2022, artificial intelligence (AI) in breast cancer, systemic therapy updates, the management of central nervous system (CNS) metastases, multidisciplinary management of ductal carcinoma in situ (DCIS), special populations, the optimization of individual-based prognostic factors, toxicity management of novel therapeutics, survivorship, and updates in surgical oncology. The best poster presentations from the symposium were highlighted in a plenary session. The key highlights of these sessions have been summarized in this conference report. 

## 2. Patient Voice

Each day of the conference opened with a presentation from a breast cancer patient talking about her cancer journey. 

### 2.1. Treatment-Free after a Clinical Trial for Metastatic Triple-Negative Breast Cancer (TNBC)

Jennifer Pogue described her experience of finding a lump in her breast during the spring of 2020, at the height of the COVID-19 pandemic. Her concerns were “dismissed multiple times” given her young age (36 years) and lack of family history of breast cancer, but Jennifer persisted. She advocated for imaging and was eventually diagnosed, 8 months after finding the lump, with de novo stage IV TNBC that had metastasized to her lungs and sternum.

Jennifer was stunned by the diagnosis and by what she read online about the prognosis (e.g., “Stage IV means there’s nothing they can do for you—you’re toast”). She was left feeling “a tornado of hysteria, grief, fear, resentfulness and depression”.

Jennifer enrolled in a 2-year clinical trial of pembrolizumab, a humanized antibody targeting programmed cell death protein 1 (PD-1). “Treatment became my job”, she said. She described with passionate gratitude several exchanges in which a clinical research nurse or oncologist gave her reason for “rebellious hope”. Jennifer’s response to therapy was excellent: within six months, her primary tumor was undetectable on computed tomography (CT) scans, her lung lesions had shrunk “to practically nothing” and stabilized, and her spine lesions were thought to have become benign. When the trial concluded, she was treatment-free.

“Encourage hope”, Jennifer advised the conference delegates. “Offer up a ‘maybe’ if it allows someone to live in hope instead of despair… If I had not had this shot at immunotherapy, and had gone straight to standard of care, I don’t think I would be here to tell this story”.

(Jennifer Pogue)

### 2.2. Realities of Navigating Breast Cancer as a Black Woman

Laura Moore, an elementary school teacher, delivered an emotional account of her breast cancer experience as a black woman. Diagnosed at age 44 with stage III TNBC, her life became a whirlwind of medical appointments and treatments. Yet, Laura’s narrative delved into the unspoken burdens that came with her experience as a racialized patient. She recounted the diagnosis making her feel like a statistic, but the under-representation in the system made her feel like “a margin within a margin”.

As a black woman, Laura unveiled the unexpected toll that treatment took on her body that was not clearly communicated to her “in any pamphlet”. From hyperpigmentation to hair loss, each transformation was an emotional journey intertwined with history and identity. 

Her struggle for inclusion and representation echoed throughout, as she advocated for change in policy, research, and healthcare practices. Laura’s survival is fuel for her commitment to those marginalized within the system, reaffirming the importance of compassionate care and diverse representation in the fight against breast cancer.

“Every week, they would ask me if I suffered any pain, redness, or peeling. And every week, I would remark that because of my melanated skin, I do not get red”.

(Laura Moore)

### 2.3. Living with Stable Metastatic Breast Cancer

Vesna Zic-Côté’s journey with breast cancer began in 2012 when, as a mother of 3 young children, she was diagnosed with stage IIB breast cancer and underwent surgery, chemotherapy, and radiation. In 2017, after 6 months of misdiagnosis of her progressively worsening symptoms (including a cough so severe it resulted in broken ribs), Vesna’s insistence on imaging eventually resulted in a diagnosis of metastatic recurrence in both lungs, the neck vertebrae, and the lymph nodes.

She was started on a new therapy, “which has held the cancer at bay to this day”, Vesna reported. She and her oncologist came to an understanding that they would use their expertise to help manage her cancer, but she would be responsible for achieving balance in her life and maintaining her physical and emotional health. To this end, the Psychosocial Oncology Unit at her cancer center has been invaluable.

Canadians living with cancer face many struggles, Vesna said. Many of the cancer patients with whom she connected online have died. Others struggle with serious side effects from both the cancer and its treatment. Yet others wait in angst, “not knowing if they will be able to try a recommended treatment that might be available, but not necessarily to them”. 

Vesna encouraged the delegates to engage patients in research and to continue pressing for urgency in the treatment of metastatic breast cancer.

## 3. Top 5 Papers in Breast Cancer 2022 (Table 1)

### 3.1. Top 5 Papers in Systemic Therapy of Breast Cancer of 2022

#### 3.1.1. POSITIVE Study [1]

This prospective single-arm cohort trial investigated whether adjuvant endocrine therapy could be safely interrupted in 516 premenopausal women (≤42 years of age) for the purposes of pregnancy. The 3-year recurrence rate was 8.9% in the treatment-interruption group vs. 9.2% in patients who did not interrupt therapy in a matched cohort analysis. Of note, 63.8% of participants experienced at least 1 live birth. This was the first study to address the impact of pregnancy on breast cancer recurrence and suggests that access to fertility preservation information and options including the interruption of endocrine therapy in low-to-moderate-risk breast cancer patients seeking a pregnancy should be standard of care.

#### 3.1.2. monarchE Study [2]

Patients with high-risk early hormone receptor-positive (HR+) breast cancer (N = 5637) were randomized to receive endocrine therapy with or without abemaciclib, a cyclin-dependent kinase 4 and 6 inhibitor (CDK4/6i), for 2 years. A significant 34% reduction in developing an invasive disease-free survival (IDFS) event was observed. This was the first study to demonstrate a significant improvement in outcomes with adjuvant CDK4/6i in high-risk HR+ breast cancer patients, suggesting that abemaciclib should be incorporated into current practice in eligible patients. 

#### 3.1.3. KEYNOTE-355 Study [3]

This study sought to determine if the addition of pembrolizumab to chemotherapy (treatment physician’s choice) improved survival in 847 patients with metastatic TNBC. In patients with a combined positive score (CPS) >10 who received pembrolizumab and chemotherapy, the median overall survival (OS) was 23 months vs. 16 months in patients who received chemotherapy alone (hazard ratio [HR] 0.73, *p* = 0.0185). These results are important for patients with metastatic TNBC (considering recent 5-year OS data for Ontario [4]) and offer new treatment options to these patients.

#### 3.1.4. TROPiCS-02 Study [5]

This study investigated whether sacituzumab govitecan (SG), a Trop-2 directed antibody (sacituzumab) and topoisomerase inhibitor (govitecan) drug conjugate, improved survival in patients (N = 543) with heavily pretreated, relapsed, or refractory hormone receptor-positive/human epidermal growth factor receptor 2-negative (HR+/HER2−) metastatic breast cancer (≥2 chemotherapeutic regimens). At a median follow-up of 12.6 months, a 1.5-month improvement in median progression-free survival (PFS) (5.5 vs. 4.0 months; HR 0.66, *p* = 0.0003) and a 3.2-month improvement in median OS (14.4 vs. 11.2 months; HR 0.79, *p* = 0.02) were observed in patients who received SG vs. those who received chemotherapy. Although the study demonstrated statistically significant benefits, side effects may impact the overall clinical impact of SG in the treatment algorithm, which highlights the importance of shared decision making between clinicians and patients.

**Table 1:** Top 5 papers in breast cancer 2022.

Study Name	Treatment	Clinical Implications
Top 5 Papers in Systemic Therapy		
POSITIVE Study	Adjuvant ET interruption for pregnancy	Access to fertility preservation should become standard of care for young breast cancer patients
monarchE Study	Abemaciclib plus ET vs. ET alone in HR+ eBC	Abemaciclib should be incorporated in current practice for high-risk HR+/HER2− eBC patients
KEYNOTE-355 Study	Pembrolizumab plus ChT vs. ChT alone in mTNBC	Offers new treatment options to improve survival in patients with mTNBC with CPS > 10
TROPiCS-02 Study	SG vs. ChT in heavily pretreated HR+/HER2–− mBC	Shared decision making is vital due to significant benefits but potential side effects of sacituzumab govitecan
DESTINY Breast 04 Study	T-DXd vs. ChT in pretreated HER2-low mBC	T-DXd is now established as a new 2L standard of care in HER2-low HR+/HR− mBC
Top 5 Papers in Radiation Therapy		
DCIS Boost	Tumor bed dose boost after WBI	A tumor bed dose boost after WBI improves outcomes for non-low-risk DCIS
Omitting Radiotherapy in Early Breast Cancer	No irradiation vs. WBI	Omitting radiotherapy for women aged 65+ with low-risk HR+ eBC does not affect distant recurrence or OS
Danish IMN Study	Internal mammary node irradiation (IMNI)	IMNI is associated with improved long-term survival in breast cancer patients
NRG-BR002 Study	SBRT plus standard systemic therapy vs. systemic therapy alone	SBRT may not provide benefit when added to systemic therapy for oligometastatic breast cancer
CURB Oligoprogression Trial	SBRT vs. standard of care for oligoprogressive disease	SBRT does not significantly prolong PFS in oligoprogressive mBC compared to NSCLC
Top 5 Papers in Surgical Oncology		
CARRIERS Study	Assessment of risk for contralateral breast cancer (CBC)	Genetic screening and risk assessment for CBC can be guided by the presence of specific pathogenic gene variants
ACOSOG Z11102 (Alliance) Study	Breast-conserving therapy (BCT) for MIBC	BCT is safe for patients with MIBC
SOUND Trial	Omitting sentinel lymph node biopsy (SLNB) in small primary breast cancer with negative US	Omitting SLNB may be oncologically safe for selected patients with small breast cancers and negative US
OPBC-04/EUBREAST 06 Study	Targeted axillary dissection (TAD) vs. SLNB in node-positive patients post-neoadjuvant chemotherapy	SLNB or TAD are adequate axillary staging methods post-neoadjuvant chemotherapy in node-positive patients
ECOG ACRIN EA2108 Trial	Locoregional treatment (LRT) vs. systemic therapy for de novo mBC	Early LRT does not improve OS or quality of life in de novo mBC, though it may improve local control

Abbreviations: 2L = second line; ChT = chemotherapy; CPS = combined positive score; DCIS = ductal carcinoma in situ; eBC = early breast cancer; ET = endocrine therapy; HER2 = human epidermal growth factor receptor 2; HR = hormone receptor; mBC = metastatic breast cancer; mTNBC = metastatic triple-negative breast cancer; NSCLC = non-small cell lung cancer; OS = overall survival; PFS = progression-free survival; SBRT = stereotactic beam radiotherapy; SG = sacituzumab govitecan; T-DXd = trastuzumab deruxtecan; US = ultrasound; WBI = whole-breast irradiation.

#### 3.1.5. DESTINY Breast 04 Study [6]

This landmark study investigated whether trastuzumab deruxtecan (T-DXd), a HER2-directed antibody (trastuzumab) and topoisomerase inhibitor (deruxtecan) drug conjugate, improved outcomes in HER2-low patients (N = 540; n = 480 HR+ and n = 60 HR−) who had previously received chemotherapy. Significant benefits in median PFS (9.9 vs. 5.1 months; HR 0.51, *p* < 0.001) and median OS (23.4 vs. 16.0 months; HR 0.64, *p* = 0.001) were observed in patients with HER2-low, heavily pretreated disease. Despite toxicities (which include the risk of interstitial lung disease [ILD]), the duration of response and improvement in outcomes were effectively doubled. This study established T-DXd as a new standard of care for the second-line treatment or beyond of HER2-low HR+/HR− metastatic breast cancer.

### 3.2. Top 5 Papers in Radiation Therapy of Breast Cancer of 2022

#### 3.2.1. DCIS Boost [7]

This paper investigated whether a tumor bed dose boost after whole-breast irradiation (WBI) improved outcomes and examined radiation dose fractionation sensitivity for non-low-risk DCIS. At 5 years, a significant decrease in local recurrence was observed in patients who received a dose boost compared to those who did not (97.1% vs. 92.7%; HR 0.47, *p* = 0.00042).

#### 3.2.2. Omitting Radiotherapy (RT) in Early Breast Cancer [8]

In this study (PRIME II trial), 1326 women were randomly assigned to receive WBI (n = 658) or no irradiation (n = 668). The cumulative incidence of local breast cancer recurrence within 10 years was 9.5% in the non-RT group and 0.9% in the RT group (HR 10.4, *p* < 0.001). However, the omission of RT had no detrimental effect on distant recurrence as the first event or OS among women 65 years of age or older with low-risk, HR+ early breast cancer.

#### 3.2.3. Danish Internal Mammary Node (IMN) Study [9]

This study presented long-term results from the Danish Breast Cancer Group IMN study cohort. The 15-year OS rates were 60.1% and 55.4% (HR 0.86, *p* = 0.007) in favor of IMN irradiation (IMNI), the 15-year risk of developing distant recurrence was 35.6% and 38.6% (523 recurrences with IMNI vs. 602 without IMNI [*p* = 0.04]), and the 15-year breast cancer mortality with IMNI was 31.7% compared with 33.9% without IMNI (*p* = 0.05). Overall, IMNI reduced the risk of distant recurrence and death from breast cancer, thereby improving long-term survival.

#### 3.2.4. NRG-BR002 Study [10]

This study investigated the benefit of standard of care (systemic therapy) with or without stereotactic beam radiotherapy (SBRT) as first-line treatment for oligometastatic breast cancer. SBRT in addition to standard systemic therapy did not improve PFS or OS compared with standard systemic therapy alone in patients with oligometastatic breast cancer within this patient population of the randomized phase II trial.

#### 3.2.5. CURB Oligoprogression Trial [11]

This study assessed if SBRT for oligoprogressive metastatic lung or breast cancer prolongs PFS or OS, and alters circulating tumor (ct)DNA profile. Patients with non-small cell lung cancer (NSCLC) experienced substantial PFS benefits from SBRT compared to the standard of care (10 vs. 2.2 months; *p* = 0.002), whereas breast cancer patients did not (4.4 vs. 4.2 months, *p* = 0.2).

### 3.3. Top 5 Papers in Surgical Oncology of Breast Cancer of 2022

#### 3.3.1. CARRIERS Study [12]

This study demonstrated that the risk of contralateral breast cancer (CBC) is increased in women with germline pathologic variants in *BRCA1*, *BRCA2*, *PALB2*, and *CHEK2*.

#### 3.3.2. ACOSOG Z11102 (Alliance) Study [13]

The purpose of this study was to evaluate oncologic outcomes in patients undergoing breast-conserving therapy (BCT) for multiple ipsilateral breast cancer (MIBC). Patients with 2 or 3 foci of biopsy-proven cN0-1 breast cancer (n = 204), most of whom were evaluated with preoperative magnetic resonance imaging (MRI), underwent lumpectomies with negative margins followed by whole-breast radiation with a boost to all lumpectomy beds. This study demonstrated that breast conservation is safe for patients with MIBC, with a 5-year local recurrence of 3.1%.

#### 3.3.3. SOUND Trial [14]

This study sought to evaluate the oncological safety of omitting sentinel lymph node biopsy (SLNB) in patients (N = 1463) with small primary lesions (unifocal cT1N0) and negative preoperative ultrasound (US) imaging. The omission of SLNB was shown to be non-inferior to sentinel lymph node staging and did not appear to alter adjuvant therapy recommendations.

#### 3.3.4. OPBC-04/EUBREAST 06 Study [15]

The rationale for this study was the lack of consensus on which axillary staging procedure should be used (targeted axillary dissection [TAD] or dual-tracer SLNB) in node-positive patients treated with neoadjuvant chemotherapy. The 3-year rate of any axillary recurrence for TAD vs. SLNB was very low (0.5% vs. 0.8%; *p* = 0.55, respectively), supporting the use of SLNB/TAD alone in node-positive patients after neoadjuvant chemotherapy.

#### 3.3.5. ECOG ACRIN EA2108 Trial [16]

In this study, 256 patients with de novo stage IV breast cancer were randomized to continue systemic therapy (n = 131) or undergo locoregional treatment (surgery and RT) for the primary site. There was no difference in OS with early locoregional therapy (HR 1.11, *p* = 0.57). Local control was significantly better in patients who received early local therapy, although this did not translate to improved quality of life.

## 4. AI and Breast Cancer

### 4.1. AI and Breast Imaging

A comprehensive overview of AI’s current and potential roles in breast imaging was presented. Deep learning-driven AI is applicable in mammography, breast US, and breast MRI, enhancing radiologist sensitivity, specificity, and efficiency.

AI’s mammography applications span lesion detection, diagnosis, triage, breast-density assessment, risk assessment, and image quality control. ProfoundAI, a commercial application, assists radiologists in identifying malignancies by assigning calibrated scores (ranging from 1 to 100) to indicate the level of confidence in prediction. 

Breast US AI encompasses lesion detection and diagnosis of lesions as benign or malignant. KiosDS, a tool generating malignancy probabilities, aids by classifying the lesion into 1 of 4 categories (benign, probably benign, suspicious, or probably malignant). 

Breast MRI AI can aid in the diagnosis of lesions as benign or malignant. Commercially available QuantX is an application that segments a user-selected lesion to generate a malignancy likelihood score based on a list of key features (such as time to peak, variance, curve shape index, washout rate, and texture). 

Current AI applications in breast imaging can triage cases to provide decision support, but lack FDA approval as a sole source of interpretation. Further research aims to investigate the application of AI in the prediction of axillary lymph node metastasis, response to neoadjuvant therapy, and risk of recurrence, as well as in the identification of imaging biomarkers.

### 4.2. AI and Pathology/Tumor Profiling

AI’s emerging role in pathology was discussed, with emphasis on the need for digital platforms as a base for effective integration. Digital pathology is already valuable in education, remote consultations, and research. AI can aid in stain analysis, biomarker assessment, workflow improvement, and diagnostics. It is employed for quality assurance, like mitotic counting, and tumor analysis. Insights were provided into an AI system’s capability to prioritize, order tests, measure size, assess grade, and prefill reports, ultimately streamlining diagnosis and saving time.

In lymph node assessment, CAMELYON16 compared algorithms and pathologists. Seven deep-learning algorithms outperformed 11 pathologists in time-limited diagnostics [17]. A recent study used 32,000 nodes from 8000 patients to train an AI program, which then demonstrated that AI assistance improved sensitivity and reduced turnaround time [18], showing that AI can identify various phenotypes. AI identified 51 features in breast cancer biopsies, aiding real-time quality control and detecting missed cancers [19].

AI’s potential encompasses prognostic prediction, therapeutic response, and gene screening. It may predict homologous recombination deficiency (HRD) status and Oncotype scores, identifying related features. It was noted that AI has evolved from quantification to diagnostics, highlighting the collaboration between AI and pathologists as “augmented intelligence”. This approach could ease pathologists’ workload and reduce diagnostic subjectivity.

### 4.3. AI and Treatment Outcomes/Real-World Data

The merging of real-world data (RWD), real-world evidence (RWE), and AI in cancer care was discussed. The importance of RWE lies in the bridging of clinical trials and real-world practice as patient populations differ and trial outcomes are extended to broader contexts in real-world practice. Regulatory bodies, clinicians, patients, and health technology assessors are increasingly recognizing the value of RWE.

Challenges in real-world cancer data due to unstructured or complex formats were highlighted, and two AI-driven strategies to address these challenges were proposed. 

Natural language processing (NLP) was exemplified in a study involving over 2000 breast cancer patients, as it effectively extracted vital patient data like Eastern Cooperative Oncology Group (ECOG) status, smoking history, and metastatic information from unstructured clinical records. However, data completeness remains an issue.

Complex structured data, like pharmacy records and patient-reported outcomes, were discussed. Machine learning algorithms were pivotal in converting intricate data into more comprehensible formats, aiding treatment categorization, determining oral/intravenous regimens, assessing dose density, and tracking therapy lines and durations.

AI’s potential in harmonizing data from NLP-based extraction to machine-learning-enhanced transformation was underscored. This integration can substantially enhance evidence-based clinical decisions, bolstering the quality of cancer care.

### 4.4. AI and Clinical Trial Accrual

In this segment, the integration of AI to enhance cancer clinical trial enrollment was discussed. A recently published systematic review and meta-analysis of ten articles involving >50,000 patients analyzed the use of AI in addressing the problem of poor patient recruitment leading to study termination [20].

AI’s role involved processing structured and unstructured data. Structured data, like age, sex, laboratory results, and staging, are readily available from electronic medical records. Unstructured data, like medical history, require NLP conversion. Classification algorithms are then applied, determining trial eligibility.

The results indicate high sensitivity (91%) and specificity (99%), reflecting effective screening potential. AI algorithms save time and outperform industry-developed solutions like IBM Watson. 

AI showed potential in identifying patients missed by human screening, although challenges remain in defining ineligibility reasons, algorithm heterogeneity, limited prospective deployment, and variations in data sets. AI’s potential in improving trial enrollment and diversifying participants is noted, yet technical and transparency concerns persist.

## 5. Breast Cancer and Systemic Therapy Updates

### 5.1. Top Clinical Advances in HR+ Breast Cancer

Important advances in HR+ breast cancer therapy were discussed. For earlier stage disease, CDK4/6is ribociclib (NATALEE) and abemaciclib (monarchE) improved clinical outcomes in high-risk stage II-III HR+/HER2-negative/low breast cancer [2,21]. For metastatic breast cancer, treatment options are expanding. The inhibition of different nodes in the *PI3K*/*PTEN*/*AKT* pathway has seen significant clinical activity, demonstrated by the addition of an *AKT* inhibitor capivasertib on top of the selective estrogen receptor (ER) degrader fulvestrant (CAPItello-291) [22].

Antibody–drug conjugates (ADCs) T-DXd (DESTINY-Breast04) and SG (TROPiCS-02) have demonstrated superior efficacy compared to chemotherapy [6,23]. However, on top of standard chemotherapy-associated adverse effects, pneumonitis/ILD is a consideration with certain ADCs. 

In terms of testing, molecular detection of resistance may lead to better long-term clinical outcomes. The PADA-1 trial demonstrated that switching to a fulvestrant backbone at the detection of an *ESR1* mutation following first-line CDK4/6i was superior to switching at clinical progression for both PFS1 and PFS2. The impact on OS is yet to be established [24].

With this progress, clinicians now have tools to better individualize treatments. However, questions remain regarding optimal treatment sequencing and access to testing and drugs. Importantly, inequitable access to therapies within Canada limits some patients from receiving the most appropriate therapies. To overcome this disparity, urgent attention is required and efforts need to be made within the context of Canada’s publicly funded healthcare mandate.

### 5.2. Advances in HER2+ Breast Cancer

Updates in neoadjuvant/adjuvant treatments for early breast cancer, treatment sequencing in metastatic breast cancer, and the role of CNS monitoring in HER2+ disease were discussed.

Global standard of care for early HER2+ breast cancer includes targeted neoadjuvant therapy with HER2-directed antibodies trastuzumab + pertuzumab on top of traditional chemotherapy. Using an adaptive positron emission tomography (PET) strategy to monitor response, the PHERGain study demonstrated that approximately 1 in 3 would respond to trastuzumab and pertuzumab alone—without chemotherapy—with no difference in survival outcomes and fewer adverse events [25]. Unfortunately, while PET is funded in some areas, there is no public coverage for pertuzumab in Canada, and therefore, this approach to tailoring neoadjuvant therapy does not currently apply to most Canadian practices. 

A proposed Canadian treatment algorithm for HER2+ breast cancer was presented, with highlighted evidence supporting the use of T-DXd following failure of trastuzumab emtansine (T-DM1), a HER2-targeting antibody (trastuzumab) linked to a cytotoxic agent (emtansine) (DESTINY-Breast02) [26] and in patients with brain metastases (DESTINY-Breast03, 01 subgroup; TUXEDO-1) [27,28,29]. Tyrosine kinase inhibitors (TKIs) such as tucatinib in combination with other therapies also have an important positive impact on brain metastases (e.g., HER2CLIMB) [30]. Furthermore, there are data suggesting that moving T-DXd and tucatinib earlier in treatment sequencing decreases the rate of receiving subsequent lines of therapy for HER2+ metastatic breast cancer. Ongoing trials are investigating their use in earlier stage disease (DESTINY-Breast09 NCT04784715, 11 NCT05113251; HER2CLIMB-05 NCT05132582).

T-DXd appears to be effective with an acceptable safety profile across age groups; however, appropriate patient selection is important and CT monitoring for pneumonitis/ILD every 6–9 weeks for the first 12 months is essential. Retreatment after Grade 2 ILD is not recommended. Additionally, while T-DXd is approved in Canada, there is no access in third-line therapy (following 2 lines of HER2-directed therapy) and beyond. 

This segment concluded by postulating that with these newer HER2-targeted therapies, “curing the incurable” is becoming possible, as there are many long-term survivors. There are significant survival gains with T-DXd and tucatinib combinations, and while toxicity is a challenge, the biggest barrier remains access, and clinicians must continue advocating for patients. 

### 5.3. Advances in TNBC

Exciting advances in TNBC over the past 5 years were discussed, signaling departure from the “only chemotherapy” era. The availability of poly (ADP-Ribose) polymerase inhibitors (PARPi), programmed cell death protein 1/programmed death-ligand 1 (PD-1/PD-L1) checkpoint blockers, and ADCs creates a need to consider subsets of TNBC. Predictors of response to treatment with pembrolizumab plus chemotherapy include PD-L1 expression levels (CPS > 10) and no prior lines of chemotherapy (KEYNOTE-355) [3]. 

Platinum-based chemotherapy is preferred in cases with a germline *BRCA* mutation (TNT trial), and PARPi are another targeted option (though not readily available in Canada due to a lack of public funding). Furthermore, ADCs have shown remarkable activity compared to standard chemotherapy, which has very little activity in TNBC (SG, ASCENT; T-DXd, DESTINY-Breast04, HER2-low subgroup) [6,31]. As discussed earlier, toxicities are chemotherapy-like with the additional consideration of pneumonitis/ILD. Ongoing investigations include emerging ADCs (datopotamab deruxtecan, BEGONIA and Tropion B-02), SG (ASCENT-03, 04), and adjuvant immunotherapy in high-risk early TNBC (pembrolizumab, KEYNOTE-522, OptimICE-PCR). An important challenge with current clinical development is that with many therapies combined, it is difficult to tease out which specific outcomes are due to immunotherapy.

Importantly, a diagnosis of TNBC is essential to access the therapies, with regulatory approvals limiting use to ER < 1%, progesterone receptor (PR) < 1%, and HER2− (i.e., not ER1+), despite ER < 10% disease often behaving (biologically and clinically) like ER-negative disease. Earlier use of novel therapies is reducing the incidence of metastatic recurrences in high-risk patients. As such, recognition of eligible patients and early referral from surgeons is important to enable access to neoadjuvant immunotherapy. Integrated multidisciplinary management for surgery/systemic therapy/pathology is critical for optimal care delivery. 

## 6. CNS Metastases

### 6.1. Radiosurgery for Brain Metastases in the Era of Targeted Therapy

An overview of radiosurgery, advances in systemic therapy active in the CNS, and current questions pertaining to the use of radiosurgery and systemic therapy together was presented.

Brain metastases have historically been treated with whole-brain RT (WBRT) and, more recently, stereotactic radiosurgery (SRS). While WBRT can reduce both local and distant brain failure, it has an impact on neurocognition. SRS is more focal, provides good local control, and is associated with less cognitive deterioration and improved quality of life [32,33], but has a high probability of distant brain recurrence. Now, HER2-directed therapies have been found to treat active and stable brain metastases with good overall responses. As such, the prognosis of breast cancer patients with brain metastases has improved, with patients in the lowest risk category achieving an OS of 3 to 4 years [34]. 

But what happens when SRS and systemic therapy are combined—are they synergistic? They may be, but the language used in radiation trials and systemic trials is different, and there are limited data on their combination. Furthermore, whether or not there is an increased risk of radiation necrosis remains a question. Risk of necrosis is associated with the dose and volume of radiation, but there are conflicting reports on whether there is increased risk with added systemic therapy. What is clear is that steroid use is associated with lower response and poorer outcomes in patients with brain metastases [35].

If SRS and systemic therapy are to be used, the ideal treatment timing and sequencing remains uncertain. Reasonable approaches may include upfront HER2-directed therapy for low-volume, asymptomatic brain metastases; upfront SRS in symptomatic patients or metastases in eloquent brain regions; and implementing a 1–2-week gap between SRS and systemic therapy.

While there are exciting advances in therapies targeting brain metastases, more studies are needed to address the effectiveness, sequencing, and toxicity of combining SRS and systemic therapy.

### 6.2. Should We Be Screening for CNS Metastases?

In this segment, clinical considerations for whether to screen for brain metastases, referencing results from a study in British Columbia, were presented. This study found that for patients with brain metastases, receiving systemic therapy after RT is an important predictor of OS. Notably, HER2-targeted therapies were most effective, followed by hormonal therapies [36].

Now that people are living longer with breast cancer, more brain metastases are seen. In the post-neoadjuvant KATHERINE study, despite the overall improvement in outcomes, 5.9% of patients with residual disease receiving T-DM1 had CNS recurrence as their first IDFS event vs. 4.3% with trastuzumab; however, for most patients on T-DM1, this was their only IDFS event, and T-DM1 was associated with a longer median time to CNS recurrence [37]. With better extracranial disease control, brain metastases are more likely to become the first IDFS event. 

Highlights of trials of HER2-targeted therapy in HER2+ metastatic breast cancer with brain metastases included a marked improvement in OS (9 months) with the addition of tucatinib to trastuzumab plus capecitabine in patients with active brain metastases (HER2CLIMB) [38]. T-DXd was shown to have benefit in stable brain metastases (DESTINY-Breast03) [27] and in newly diagnosed patients or those progressing after local therapy (TUXEDO-1, DEBBRAH cohorts 1–3) [29,39]. There are more limited data in TNBC. KEYNOTE-355 demonstrated OS improvement with pembrolizumab overall but included a very small number of patients with brain metastases (none active) [3], while an ASCENT subgroup analysis demonstrated modest improvement in brain metastases with the ADC SG [40]. Interestingly, KEYNOTE-522 demonstrated that achieving pathological complete response (pCR) is associated with excellent outcomes, which are significantly worse in the presence of residual disease [41]. Adjuvant immunotherapy offers benefit in TNBC, but a high risk of relapse remains.

Screening for brain metastases may be justified in metastatic HER2+ disease and TNBC. European guidelines state that screening at diagnosis is potentially justified in metastatic HER2+ and TNBC, if detection alters the choice of systemic therapy [42,43]. MRI with contrast is a favored approach for screening (suggested every 6–12 months but with no consensus). However, given that MRIs can be anxiety-inducing, some feel that a randomized controlled trial (RCT) demonstrating improved quality of life and OS is necessary before changing practice. A risk-adaptive screening strategy may also be considered, such as screening patients based on dynamic markers of response to systemic therapy (e.g., ctDNA). 

Screening for brain metastases could allow for earlier localized RT and initiation of systemic therapy, while delays could subject patients to more treatment and toxicity. Patients with active CNS metastases have often been excluded from trials, and healthcare providers and patient advocates should strive to change this. 

## 7. Multidisciplinary Management of DCIS

### 7.1. Management of DCIS Today—The Radiation Oncology Perspective

Background on RT in the management of DCIS was provided and current best practices were reviewed. The advent of broad mammography screening has dramatically improved DCIS detection. While mastectomy is very effective, BCT is preferable from psychological and quality of life perspectives. The addition of RT to BCT helps to prevent local recurrence across the spectrum of disease risk, and partial breast irradiation may be appropriate for some low-risk patients. Boosting RT among high-risk patients may provide a benefit to local recurrence but increases the risk of breast pain and induration. Overall, data support RT escalation in high-risk lesions and de-escalation in lower risk scenarios. It is hoped that future research will confirm the effectiveness of ultrahypofractionated RT protocols currently being explored for DCIS. 

### 7.2. Molecular Profiling to Guide Radiation Therapy

The current state of molecular profiling in risk stratification and its role in DCIS treatment was described. Earlier detection of disease improves outcomes in breast cancer, but also detects tumors that will never become clinically evident. While DCIS is not usually fatal, it can develop into an invasive disease with a significant mortality rate. However, there are currently no predictors of invasive recurrent disease to address concerns about “overtreatment” of lesions that will never become invasive.

Current research on multigene signatures and predictive modeling hopes to improve the stratification of risk. The 12-gene DCIS Score multigene signature has some ability to identify low-risk patients who may not benefit from RT, but other genotyping arrays require further external validation. Combining the 12-gene DCIS Score with tumor size and age into a predictive model seems to provide insight into risk at the individual level, a strategy that is currently being evaluated in a prospective study (ELISA, NCT04797299).

### 7.3. Systemic Therapy for DCIS

Key milestones in the use of systemic therapy for reducing recurrence risk after treatment for DCIS were described. Both tamoxifen and aromatase inhibitors improve long-term prevention of recurrence in breast cancer. Of note, the TAM-01 (Baby-Tam) study treated patients with intraepithelial neoplasia (69% with DCIS) with 5 mg/day tamoxifen for 3 years after surgery with or without RT. After a 10-year follow-up, the risk of recurrent invasive cancer or DCIS in the overall population was reduced by over 40%, and the risk of CBC was reduced by 64%. In the DCIS cohort, there was a 50% reduction in breast cancer events [44]. Women taking tamoxifen experienced an average of 1 more hot flash per day, with no other significant increases in adverse events. 

Clinically, physicians should weigh the evidence of the TAM-01 study against other studies of tamoxifen, given its relatively low enrollment of 500 patients; and note that 5-mg tablets are not available in most jurisdictions. 

### 7.4. Considerations for BCT after Local Recurrence

In this segment, approaches to local recurrence after treatment for DCIS were described. Total mastectomy is the recommended approach for local recurrence after an initial treatment with breast-conserving surgery (BCS) and RT, but repeat BCS and RT may be considered for some patients. There seems to be little difference between salvage mastectomy and a second conservative treatment with respect to 10-year disease-free survival (DFS) or OS. Partial-breast irradiation is preferable over WBI to mitigate toxicity. Preliminary evidence suggests that hypofractionation protocols may not compromise effectiveness, and a study (PRESERVE NCT05592938) is currently underway to evaluate their feasibility in local recurrence. 

## 8. Special Populations

This session focused on patient populations that are often under-represented in clinical trials. The aim was to bring attention to disparities that can potentially impact a patient’s experience throughout their cancer journey.

### 8.1. Breast Cancer and Older Adults

Breast cancer predominantly affects older adults, with an average age at diagnosis of 63 years; and. older individuals account for >50% of breast cancer-related fatalities. Aging introduces significant challenges related to cancer care, including frailty, mobility limitations, compounding comorbidities, and polypharmacy among others. These can all impact tolerance of, and adherence to cancer therapies, and complicate the risk-benefit assessment. 

The integration of age-related predictive tools can facilitate treatment decision making. E-prognosis (https://eprognosis.ucsf.edu/, accessed on 25 October 2023) is an online tool that helps estimate patient life expectancy based on a number of relevant factors, such as age, gender, independence, mobility, self-rated health, hospitalization history, and comorbidities. The Cancer and Aging Research Group–Breast Cancer (CARG-BC; https://www.cancercalc.com/carg.php, accessed on 25 October 2023) tool uses both disease- and age-related factors to assess the potential of grade 3–5 chemotherapy toxicities. It has been validated specifically in patients aged 65 and older [45].

Although standard cancer care options can be equally beneficial in the older patient population, age-specific competing risks to health and life as well as therapeutic windows of available treatments must also be fully considered to optimize outcomes. 

### 8.2. Young Women and Breast Cancer

This section opened with findings from the ongoing RUBY study (https://www.rubystudy.ca/Home), which is investigating breast cancer in women aged 40 or younger—a patient group with distinct treatment challenges. Young patients often present with more aggressive tumors and face poorer outcomes exacerbated by patient- and system-related diagnostic delay. Contributing factors include lack of awareness, ineligibility for screening programs, low pretest probability of cancer, non-specific breast complaints, and a high rate of benign breast disease. 

Findings from RUBY, conducted across 33 Canadian sites, revealed that 89% of participants presented with a breast lump at diagnosis, with pain being a surprisingly common symptom. Furthermore, 20% had actionable genetic mutations, emphasizing the need for expansion of genetic testing in young women. The study also highlighted the psychosocial impacts of treatment, body image concerns, fertility, and sexual health issues. 

Efforts at enhancing oncofertility education for surgeons and patients often result in increased referrals for fertility preservation. Begin Exploring Fertility Options, Risks, and Expectations (BEFORE https://fertilityaid.rethinkbreastcancer.com, accessed on 25 October 2023) is a patient tool to aid in fertility discussions. Furthermore, theand the importance of congruence in decision-making in enhancing patient satisfaction, emphasizing the role of patient preferences cannot be understated. An app, developed in partnership with Headversity, aims to provide peer support and track patient well-being in the full RUBY cohort. 

### 8.3. Pregnancy and Breast Cancer

This session included a comprehensive overview of breast cancer during pregnancy. Recent findings by Cairncross et al. published in the *Journal of the American Medical Association (JAMA) Oncology* revealed lower survival rates in pregnancy-associated cancers compared to non-pregnant individuals, with time to diagnosis and first treatment as contributing factors [46]. The database entitled Mothers and Babies: MBRRACE-UK from 2020 revealed four key maternal mortality factors, including cardiac events linked to assisted reproductive technologies, confusion with mastitis, staging deficiencies, and delays in third-trimester chemotherapy.

A recent Canadian prospective comparative study reported no fetal neurotoxic effects following prenatal chemotherapy exposure. Maternal chemotherapy, the use of imaging tools, and surgery exhibited minimal teratogenicity post-first trimester, given the completion of organogenesis. Attention should be paid to monitoring growth restriction, placental dysfunction, and preterm labor [47].

The multidisciplinary approach of the Pregnancy-Associated Breast Cancer Program offers insights into pregnancy care, investigations, surgical considerations, chemotherapy administration, birth planning, and post-partum care. Accurate pregnancy dating is crucial for chemotherapy and surgery planning. Screening methods like enhanced first trimester screening and non-invasive prenatal testing are available, with diagnostic options such as amniocentesis and chorionic villus sampling if needed. Patient preference regarding termination options, including medication, surgical abortion, and labor induction, should be discussed during prenatal counseling. A comprehensive care pathway was introduced for breast cancer management throughout pregnancy, facilitating vigilant monitoring.

### 8.4. Racial Disparities

In this segment, health was defined as complete well-being and equity as the absence of systemic differences impacting the breast cancer journey. Thereby, health equity should be defined as everyone having equal opportunity to reach their health potential. 

Racial disparities have been observed to impact breast cancer outcomes; examples of contributing factors include differential exposure to risks, social stratification, socio-economic disparity, and unequal allocation of power and resources leading to differential access to health services. Two studies were explored: one revealing lower screening rates among recent immigrants to Canada, and another highlighting disparities in genetic testing and subsequent surgical interventions among racial subgroups. Factors contributing to suboptimal care included health literacy, distance to health clinics, access to medical genetic expertise, and practitioners’ values and likelihood to recommend screening and genetic testing to their patients. 

A study by Dunlop et al. in *JAMA* investigated whether the representation of racial groups in 221 patients who completed phase I trials was proportional to the incidence of cancer in those racial groups as determined through the Cancer Incidence in North America (CiNA) database [48]. The findings revealed an over-representation of white patients and under-representation of other ethnic groups, and these reported differences increased with time. The need for ongoing research and interventions to address these disparities was emphasized.

## 9. Optimization Strategies for Individual-Based Prognostic Factors

### 9.1. Pathology Update on Breast Biomarkers

This segment included an update on prognostic markers in breast cancer management through a multidisciplinary perspective. The three key prognostic biomarkers highlighted were grade, Kiel protein 67 (Ki-67), and recurrence scores.

The importance of accurate prognostic assessment in guiding treatment decisions for optimal patient care was emphasized. Grading, particularly mitotic count, offers insights into tumor behavior and patient prognosis. Ki-67, a protein linked to cell division, is examined through immunohistochemistry (IHC) to determine the proliferative rate of cancerous cells, aiding in prognosis determination. Additionally, gene expression profiling, through polymerase chain reaction (PCR) nano-string or RNA sequencing, provides an Oncotype recurrence score used for its predictive value in assessing the likelihood of disease recurrence. The Magee Decision Algorithm^TM^ can be used to triage cases for molecular testing [49].

Specimen handling is an important part of biomarker testing. Formalin-fixed biopsies are best used for gene expression analysis to minimize RNA degradation and ensure reliable test results. Unlike RNA, protein fixation is better preserved over time; therefore, protein biomarkers (Ki-67, ER, HER2) can also be assessed on older biopsies. Time from surgery to laboratory preparation should be minimized as the mitotic activity of samples can be affected by ischemia. Clinicians should also consider whether the test results are interpreted manually by pathologists or automated by computational analysis that may miss nuanced features of tumor samples. Finally, biomarker testing is more reliably performed on biopsy specimens (rather than resections) due to the higher surface-area-to-volume ratio.

### 9.2. The Role of Neoadjuvant and Adjuvant Therapies

This segment discussed the integration of laboratory prognostic analyses in clinical decision making. Various prognostic factors influence the risk of recurrence, including tumor size, lymph node involvement, grade, Ki-67, genomic scores, neoadjuvant therapy response, age, and premenopausal status. Notably, achieving pCR post-neoadjuvant therapy improves event-free survival (EFS). Neoadjuvant chemotherapy is most appropriate for locally advanced and inflammatory breast cancer, with TNBC and HER2+ cases showing higher pCR rates. Tailoring adjuvant therapy based on residual disease optimizes outcomes.

Predictive assays (Oncotype DX, MammaPrint, and Ki-67) guide chemotherapy and endocrine therapy decisions to reduce recurrence risk in high-risk patients (such as using Ki-67 staining in grade II patients). The monarchE and NATALEE trials also used Ki-67 and Oncotype DX scores to identify high-risk patients for adjuvant CDK4/6is and reported improvements in IDFS [2,21]. Endocrine therapy, including aromatase inhibitors, enhances DFS, extending to premenopausal patients with ovarian function suppression. Shared decision making and risk assessment discussions with patients are emphasized, with the aid of tools such as the CTS5 calculator and UK Predict guiding therapies. 

### 9.3. The Surgical Approach to Nodal Disease and Margin Status

This segment discussed strategic approaches to nodal disease and margin status, highlighting that it should begin at the diagnostic evaluation stage informed by axillary US examination and tumor characterization of receptor status. The American Society of Clinical Oncology (ASCO) and Cancer Care Ontario guidelines advise against preoperative axillary US staging for clinically node-negative early-stage breast cancer (T1 and T2) to minimize unnecessary axillary dissections. 

Balancing over- and under-treatment, the ACOSOG Z0011 trial results revealed comparable DFS, OS, and local recurrence after 10 years in clinically node-negative patients with 1 or 2 positive nodes who underwent axillary dissection [50]. Further ASCO guidelines recommend neoadjuvant systemic therapy for HER2+, TNBC, and T1c patients to guide adjuvant therapy. 

In upfront surgery, axillary lymph node dissection (ALND) is unnecessary for BCS with 1 or 2 positive nodes, supported by the AMAROS trial [51]. Extra-nodal extension (ENE) of >2 mm strongly predicted 4 or more additional positive nodes at ALND. Axillary recurrence, especially with ENE < 2 mm, was minimal.

Managing positive margins involves oncoplastic surgery, innovative technologies like pegulicianine fluorescence, and detailed operative notes for margin assessment. Breast MRIs aid multifocal/multicentric disease detection.

Clipped node retrieval in 4 neoadjuvant trials (SENTINA, SN FNAC, Alliance ACOSOG Z1071, and GANEA 2) reduced false negatives after neoadjuvant chemotherapy, supporting the National Comprehensive Cancer Network (NCCN) guidelines. Optimal negative margin width after neoadjuvant chemotherapy remains debated; NSABP B-18 and B-27 trials employ “no ink on tumor” margins for superior control rates. 

Parallel axillary strategies apply to neoadjuvant endocrine therapy. Ongoing trials delve into nodal status and margin strategies in neoadjuvant systemic therapy, involving ALND for residual disease, addressing residual calcifications and DCIS, emphasizing the imperative of prospective trials for surgical strategy refinement.

### 9.4. Considerations for Radiation Therapy

The segment opened with a complex case involving a premenopausal woman with node-positive breast cancer. The importance of ruling out pregnancy before adjuvant RT was emphasized, with advocacy for family history assessment for potential medical genetics referrals and tumor analysis. Mastectomy may be considered in young women; however, population-based data from British Columbia showed no apparent survival disparity between mastectomy and BCS. A radiation boost, involving 4 or 5 days of additional radiation to the tumor bed, can enhance local control post-lumpectomy, especially for women under 50 years of age. Surgical clips at the time of surgery are helpful to localize seroma for boost, especially with increasing use of oncoplastic surgery. Despite benefits of radiation boost, cosmesis should be considered, and boost may not be as beneficial in the modern era of tamoxifen and trastuzumab. Positive margins correlate with a twofold increase in breast tumor recurrence risk, not mitigated by favorable biology, endocrine therapy, or radiation boosts. Regional nodal irradiation (RNI) plays an important role in locoregional control, especially in the era of SLNB alone without completion of axillary dissection, but risks include pneumonitis, shoulder stiffness, lymphedema, and brachial plexopathy. Thus, optimizing use of RNI is an active area of research. The ongoing RHEAL trial (NCT04228991) assesses 1-week vs. 3-week dose fractionation. It was revealed that using 5 fractions for nodes remains experimental. Finally, The ongoing randomized MA.39 trial (NCT03488693), which examines regional RT in biomarker low-risk, node-positive breast cancer patients, was highlighted. The trial aims to reduce both short- and long-term morbidity while also avoiding over-treatment and cutting healthcare expenses.

## 10. Toxicity Management of Novel Therapeutics

### 10.1. Nausea, Vomiting, and ILD with ADCs

There are currently over 160 ADCs in clinical development for cancer treatment. Three have been approved by Health Canada for the treatment of breast cancer: T-DM1, T-DXd, and SG. These vary in their pharmacokinetics and pharmacodynamics (e.g., drug-to-antibody ratio and bystander effect) and in their toxicity profiles. 

The favorable toxicity profile demonstrated with T-DM1 in the EMILIA [52] and TH3RESA [53] trials created an expectation among oncologists and patients that other ADCs would have similar toxicity profiles. However, in the DESTINY-Breast03 trial, T-DXd was associated with surprisingly high levels of nausea and vomiting, decreased appetite and weight loss, and alopecia [54], and in the TROPiCS-02 study, which involved heavily pretreated patients, SG was associated with high rates of neutropenia, anemia, diarrhea, and alopecia [55]. It was stated that setting patient expectations, particularly those patients at high risk of emesis, is important. 

T-DXd use is also associated with ILD, as demonstrated in the DESTINY-Breast04 trial [6]. Careful screening and monitoring, and prompt, appropriate use of systemic steroids are essential for the management of ILD. It was reported that a Canadian position paper on the clinical management of ILD is in development. That paper has since been published [56]. 

### 10.2. Management of Radiation-Related Toxicities for Patients Undergoing Immunotherapy and Surgery

This segment discussed the Montreal University Hospital Center’s (CHUM) current approach to the management of radiation-related toxicities in patients for whom immunotherapy and surgery are planned. 

Based on the center’s participation in the KEYNOTE-522 study, it was found that adding pembrolizumab in the neoadjuvant phase does not impact surgical outcomes, does not result in delays to surgery, and does not result in any new immune-related adverse events. This reflects what was reported in the CheckMate 816 and KEYNOTE-617 trials in NSCLC [57,58]. It was also found that current or sequential treatment with pembrolizumab does not impact radiation-induced adverse events or surgical site complications. 

Because of the risk of adrenal insufficiency with this treatment approach, the CHUM measures blood AM cortisol at the initiation of treatment and again before surgery in patients receiving pembrolizumab with neoadjuvant chemotherapy. The CHUM also screens for myocarditis through routine troponin surveillance (serial troponin measurements taken weekly for 8 weeks, then every treatment cycle thereafter).

### 10.3. Cancer-Therapy-Related Cardiovascular (CV) Toxicity

This segment underscored the importance of identifying and protecting patients at risk of cancer-therapy-related CV toxicity and optimizing CV health in survivorship. Per the 2022 European Society of Cardiology (ESC) guidelines on the topic [59], strategies to this end include the following: undertaking a baseline CV toxicity risk assessment; encouraging CV-protective lifestyle changes (e.g., smoking cessation and active living); working with a cardiologist to ensure guideline-recommended medications for primary and secondary reduction of CV risk (e.g., beta-blocker, statin, angiotensin-converting enzyme inhibitor [ACEi], or angiotensin-receptor blocker [ARB]); and minimizing the use of cardiotoxic drugs. High-risk patients may require more frequent cardiac monitoring. 

The segment concluded by noting that there is a dearth of research on optimizing CV outcomes in cancer survivors and that collaboration between oncologists, cardiologists, and primary care providers is clearly needed.

### 10.4. Surgical Prevention and Treatment of Lymphedema

This section provided a review of the surgical management of lymphedema. Surgery can be used preventively, as with the lymphatic microsurgical preventative healing approach (LYMPHA). Surgery is also used for the treatment of stage III lymphedema when combined physical therapy and complex decongestive therapy (manual lymphatic drainage and compression) prove insufficient. Staging and imaging determine the appropriate surgical technique. Options include lymphaticovenous bypass (a microsurgical anastomosis of the obstructed lymphatic vessels to small, superficial veins, which allows lymph fluid to be shunted into the venous system) and lymph node transfer (which promotes lymphangiogenesis, ultimately resulting in new connections between the proximal and distal lymphatic vessels). 

## 11. Survivorship

### 11.1. Radiology Screening 101: Breast Imaging Updates

A comparison of Canadian and American breast cancer screening initiatives was discussed. Canadian task force breast cancer screening guidelines recommend screening every 2 years from age 50 for average risk individuals. In 2023, the American preventative task force recommended starting at age 40. The Canadian task force has not adopted this guidance, noting that the benefits of earlier screening does not outweigh the risks of false positives. This decision stems from issues with the CNBSS1 and CNBSS2 studies [60,61]. Critics have pointed out several problems with these studies, such as poor methodology (e.g., non-blinded randomization), the inclusion of symptomatic women in the mammogram arm, and low-quality imaging. Because of these limitations, the studies are not considered statistically reliable. 

A recent study using data from Statistics Canada showed that provinces with earlier screening have a lower incidence of stage III cancers, and a higher incidence of detection of stage I cancers [62]. Regions that do not have early screening in place or plans to do so include Northwest Territories, Nunavut, Ontario, and Newfoundland. Challenges accessing primary care also limits the proportion of qualifying patients who receive screening. As such, current lobbying recommends that qualifying patients can self-refer for screening.

Other recent changes to breast cancer screening include expanding the genetic screening requirements in Ontario, leading to a larger qualifying population. Advocacy for the use of MRI for screening in cases of dense tissue is ongoing. 

Guidance on screening in transgender patients has historically been limited. Current guidelines for transgender males recommend following the same recommendations as cisgender females if native breasts are present or if residual breast tissue is found after a mastectomy or reduction mammoplasty. Current recommendations for trans women are to begin screening following recommendations for cisgender women when gender-affirming hormone therapy has been used for more than 5 years.

### 11.2. Survivorship and Surveillance—A Clinical Case

A clinical case was described: a 50-year-old, low/average risk, post-menopausal female who was referred for assessment of breast pain and known bilateral breast cysts. Mammogram and breast US showed scattered cysts bilaterally. With 1 cyst on each side being monitored, the patient was recommended to follow up in 1 year for repeat imaging. At the follow-up, her breasts were heterogeneously dense. A mammogram showed a new irregular mass with distortion adjacent to the cyst originally being monitored. US showed 2 prominent right level I axillary lymph nodes with cortical thickening. Staging MRI showed a mass spanning almost 6 cm. Pathology of the biopsy showed invasive carcinoma with squamous differentiation.

While awaiting treatment decisions, the importance of considering all aspects of the patient’s life was noted. This patient was a single mother with limited family in Canada, but a strong support network of friends and a church community. Prior to her breast cancer diagnosis, she was considering hormone therapy for vasomotor symptoms of menopause. She was concerned about how the cancer progressed so quickly in 13 months. She took a leave of absence from work for her cancer treatment and had financial concerns about supporting her family, as well as the potential side effects from treatment. She was also concerned about reducing her risk of recurrence and what screening would be available to her going forward. As patients navigate breast cancer diagnosis, treatment, and remission, it is important to consider the nuances and challenges they face and how we can provide support across the survivorship phase of their journey.

### 11.3. Sexual Health and Genitourinary Symptoms of Menopause

Medical illness, stress, fatigue, body image, medications, certain surgical procedures, and pain are all factors affecting female sexual dysfunction in breast cancer. Genitourinary symptoms of menopause (GSM) (e.g., burning, irritation, dysuria, and painful intercourse) are present in 35–91% of breast cancer survivors; more than those without breast cancer. 

The initial management of sexual dysfunction includes validating symptoms and their effect on quality of life, discussion of options (pharmacological and non-pharmacological), referral to other supportive healthcare providers, such as therapists, and shared decision making with the patient and oncology team. Non-hormonal options are the same as those for patients without breast cancer (e.g., moisturizers and pelvic physiotherapy). 

Second-line therapy in breast cancer patients includes vaginal estrogen. Generally, systemic estrogen levels remain in the physiologic postmenopausal range with local estrogen use. Prasterone is a newer medication that is a dehydroepiandrosterone (DHEA)-inactive precursor that is converted to estrogen and androgen in the vagina. Systemic levels of estrogen after use are on the lower end of the physiologic postmenopausal range, and it is hypothesized that concurrent use of aromatase inhibitors may prevent systemic conversion to estrogen. Ospemifene, another new medication, is a selective ER modulator; it has agonistic effects in the vagina and bone and antagonistic or neutral effects in breast tissue. Studies of ospemifene have not shown an increased risk of endometrial or breast cancers [63,64,65,66]. Vaginal laser therapy has conflicting evidence in GSM and limited safety data and is not currently recommended by the Society of Obstetricians and Gynecologists of Canada/Centers for Medicare and Medicaid Services (SOGC/CMS).

### 11.4. Lymphedema and Brain Fog

This segment discussed how the onset of cancer can impact lymphedema and cognitive impairment. Breast-cancer-related lymphedema is chronic swelling that occurs as a result of lymph buildup due to damage to the lymph vessels or nodes caused by cancer treatment, cancer spreading to the lymph nodes, or significant injury. Risk factors for lymphedema include receiving cancer treatments, metastases to lymph nodes, having a history of skin infections, being overweight/inactive, or having a history of chronic swelling. Early signs include symptoms of heaviness, tightness, numbness, or pain in the arm; swelling in the upper extremities, breast, chest, trunk, or back; and prior skin infections, cording, or seroma. Lymphedema has a significant impact on patient well-being, including physical concerns (mobility, pain, and fatigue), psychosocial concerns (body image, anxiety, and frustration), and occupational performance barriers (cost of treatments, and impact on home and work life). Management of lymphedema includes physiotherapy and complete decongestive therapy (manual lymph drainage, compression, exercise, and meticulous skin care), with microsurgery used in select cases.

Cancer-related cognitive impairment (brain fog) has been documented in studies using patient reports as well as objective tests. A meta-analysis of cognitive impairment found that there was no deficit compared to controls in visual and spatial perception and processing [67]. Cancer patients, with or without a history of chemotherapy, performed worse than controls on processing speed, language, attention, memory, and executive function. Cancer patients who received chemotherapy performed worse than cancer patients who had not received chemotherapy on attention and executive function. These impairments were significant but mild; severe symptoms should involve further investigation for alternative diagnoses [67]. A meta-analysis of MRI data found that patients who received chemotherapy had an area in the right frontal lobe and the left parietal lobe that had lower activation [68]. These areas are part of the frontoparietal attention network, which is important for tasks including sustaining attention, complex problem solving, and maintaining and manipulating information in the working memory. Factors that facilitate neuroplasticity and therefore improvement in these areas include sleep, stress management, physical exercise, and a healthy diet. Behaviors that can improve cognitive function in these areas include continuous learning, social interactions, and trying new activities.

### 11.5. Surveillance and Bone Heath While on Endocrine Therapy

In this segment, the effects of endocrine therapy on bone loss were discussed. Bone loss is a common effect of breast cancer treatment. The risk of bone loss increases after menopause as estrogen, a key regulator in bone homeostasis, decreases. In addition, some therapies for breast cancer (e.g., tamoxifen, aromatase inhibitor, and ER down-regulators) directly affect bone formation. As recommendations for the duration of aromatase inhibitors have increased up to 10 years, the negative effect on bone formation and fracture risk has also increased. 

When initiating aromatase inhibitors, the patient’s fracture risk should be assessed. Exercise and optimization of calcium and vitamin D intake should be ensured with all patients, and patients at higher risk for fracture based on T-score and other risk factors should receive bone-directed therapy. Bone mineral density should be assessed after 12–24 months of bone-directed treatment. Bone-directed therapies include zoledronic acid, risedronate, and denosumab. Zoledronic acid has been shown to reduce the incidence of recurrence of cancer in bone by 34% and breast cancer-specific mortality by 17% in postmenopausal breast cancer patients. As a result, zoledronic acid is the treatment of choice in patients at high risk of recurrence or bone metastases.

### 11.6. The Microbiome and Dietary Considerations

In this segment, intermittent fasting was discussed. This diet plan, which includes limiting the times or days when a person eats, has been connected to several health benefits in the media. It has been associated with metabolic changes such as increased breakdown of fat (lipolysis) and production of ketone bodies, leading to reduced insulin spikes and a potential decrease in hunger. Moreover, intermittent fasting has been demonstrated to lower inflammatory markers and may have implications for cancer. 

A study of caloric restriction in rhesus monkeys was shown to reduce the incidence of cancer, CV disease, diabetes, and insulin resistance. A study conducted on mice with tumor cells demonstrated that intermittent fasting significantly reduced tumor sizes compared to control mice. This study further showed a link between the gut microbiome and increased intermittent fasting [69]. However, these data have not translated to human models. While a study of people observing Ramadan showed an increase in microbiome diversity after fasting, a systematic review of intermittent fasting in breast cancer patients did not find robust evidence to recommend this diet [70]. A meta-analysis of intermittent fasting compared to usual diets for weight loss did not find a significant difference [71]. Recommendations should focus on making healthier choices, such as limiting sugar and processed foods and increasing vegetables, fiber, and whole foods. For success, it is important to make recommendations in the context of the patient’s culture and foods that are part of that culture.

## 12. Surgical Oncology

### 12.1. Update on Breast MRI

Although MRI is the highest-sensitivity test available for breast cancer detection, the use of MRI for the staging and surgical planning of breast cancer remains controversial [72,73]. The evidence for the use of preoperative MRI was reviewed. The use of MRI is on the rise in clinical practice, as evidenced in an analysis of the Surveillance, Epidemiology, and End Results (SEER) database (from 0.8% in 2000–2001 to 25% in 2008–2009) [74] and in a Canadian population-based study (from 3% in 2003 to 24% in 2012) [75]. 

MRI exhibits better correlation with true pathologic sizing in invasive ductal carcinoma (IDC) than mammography or US (0.75–0.80 vs. 0.26–0.76 and 0.57–0.68 for MRI vs. mammography and US, respectively) [76,77]. Kuhl et al. demonstrated significantly improved preoperative MRI sensitivity over mammography and US for the detection of DCIS [78]. Furthermore, the concordance rate for preoperative MRI with pathology in invasive lobular carcinoma (ILC) was also superior to mammography and US [79]. The benefits of preoperative MRI were also demonstrated in ipsilateral lesions (multifocal and multicentric disease) and CBC [80,81,82]. 

Currently available data on the benefits of preoperative MRI in reoperation rates are unclear [83]. However, recent treatment guidelines from Ontario suggest that preoperative MRI does reduce reoperation rates [84]. The benefits of preoperative MRI on local recurrence and CBC rate were demonstrated in retrospective studies using propensity score matching [83]. A minimal benefit for preoperative MRI was observed for cause-specific survival and OS, although this was not statistically significant [84]. 

Despite all of the positive data, there are drawbacks to preoperative MRI. Increased rates of mastectomies have been reported, although caution is warranted when interpreting these results as there are several mitigating factors [84]. In addition, false-positive MRI findings result in increased mastectomy rates, making the case for additional biopsy proof of more extensive disease [80,85]. It was suggested that the increased mastectomy rate associated with MRI was attributed to treatment guidelines, citing German guidelines and results from the ACOSOG Z11102 trial [86,87,88]. Lastly, preoperative MRI can be associated with increased wait time and increased costs [89,90,91].

Overall, preoperative breast MRI can enhance surgical planning, improve local control, and improve survival in certain patient subgroups, although some precautions should be taken. 

### 12.2. What Is New in Oncoplastic Surgery?

Recent developments in procedure selection, techniques, and strategies to deal with complications in breast cancer surgery were reviewed. In 2019, the American Society of Breast Surgeons (ASBS) developed a consensus statement on oncoplastic surgery, which included inferior/abdominally based and lateral/axillary-based volume replacement flaps [92]. For example, the anterior lateral intercostal artery perforator (LICAP) flap is an option for direct oncoplastic breast reconstruction following lumpectomy [93]. Extreme oncoplasty describes breast conservation for patients who traditionally would have been candidates for mastectomy (e.g., T3+ tumors and multicentric disease), although communication with patients and a mastectomy plan are paramount [94]. 

Patient (e.g., breast size and density, comorbidities, and esthetic outcome) and tumoral (e.g., size, location, and skin resection) factors typically determine which procedure a surgeon will select. Once a procedure has been identified, surgeons may opt to work independently or with a colleague (i.e., plastic surgeon) based on their level of comfort, surgical planning, and logistics [95]. Certain complications, such as T-junction dehiscence, nipple necrosis, standing cone, skin necrosis, positive margins, and nipple malposition, may occur during oncoplastic surgery. Simple solutions for each were outlined [96,97].

### 12.3. The Aesthetic Mastectomy

In an aesthetic mastectomy, performed after one or both breasts are removed, remaining tissue is tightened so the chest wall appears flat. This segment reviewed the technique and trends, introduced the “Going Flat” movement, and outlined surgical considerations.

The “Going Flat” movement was led by patients who chose not to undergo reconstructive surgery following a mastectomy. Conversely, “flat denial” is associated with surgeons who advise against not reconstructing, do not offer the option of not reconstructing, or leave excess tissue to facilitate future reconstruction. A 180-degree shift was described—the number of mastectomies performed used to be a quality indicator whereas now surgeons tend to push breast reconstruction without considering their patients’ preferences. Discussing surgical options with patients is important. A survey of 931 patients who opted for aesthetic flap closure revealed that 22% of them experienced flat denial, the strongest predictor of dissatisfaction with surgical outcome [98]. 

Recent mastectomy data are not readily available. Between 2007 and 2014, 11.5% of patients in Ontario underwent immediate breast reconstruction (IBR) after a mastectomy [99]. In Alberta, between 2016 and 2018, 42% of patients underwent a mastectomy, of whom 23% opted for IBR [100]. Mastectomy and reconstruction rates increased by 146% between 2005 and 2010 in the US, where 49% of patients underwent a mastectomy (with or without reconstruction) [101]. 

It was suggested that when performing an aesthetic mastectomy, surgeons need to consider incision type and placement, managing “dog ears”, and preventing an inferior ridge. While an inframammary fold (IMF) incision is more cosmetically appealing, factors such as tumor location, patient morphology, and incision origin need to be considered. In most patients, a truly flat closure can be obtained by removing the soft tissue between the breasts, and several techniques are available to reduce dog ears, including advancement flap [102] and modified M plasty [103]. The IMF can be obliterated by continuing the incision along the upper abdominal wall, removing excess skin and inferior tissue, and using a negative pressure dressing while reminding patients that revisions may be required. 

## 13. Best Poster Presentations

### 13.1. Local and Regional Management of the Axilla in Node-Positive Breast Cancer Patients Following Neoadjuvant Chemotherapy: An Evaluation of Real-World Practice

Axilla status is important for staging, prognosis, and determining adjuvant treatments; however, comes with peri- and post-operative risks. Trials have shown no survival benefit with ALND compared to SNB and it is not known how to manage post-neoadjuvant patients with positive SNB. Current recommendations are to restage the patient and, if positive, perform ALND followed by regional nodal radiation.

A population-based retrospective study of women with node-positive breast cancer following neoadjuvant chemotherapy was performed to identify practice patterns in Alberta, measure rates of complete ALND (cALND) and breast cancer recurrence within a subgroup of patients with SNB, and identify predictors of both outcomes.

The study population had primarily unifocal, T1/T2 tumor size and pre- and post-neoadjuvant chemotherapy. Most patients had HR+ tumors and 48% were high risk (HER2+ or TNBC).

Of the patients with a positive SNB, 22% underwent cALND. After a median of 17 months, 18.8% recurred (87.9% distal, 9.1% isolated local, and 3% isolated regional). Of patients with regional recurrence, 57% had already undergone cALND. Predictors of recurrence were low body mass index (BMI), triple-negative status, and clinical T3/4 disease before neoadjuvant chemotherapy. There were variable practice patterns in Alberta and no significant association between omission of cALND and regional recurrence.

### 13.2. Reflexive Genomic Assays on Breast Care Biopsies: A Single-Center Experience

Genomic assays characterize breast cancer more accurately than traditional histopathologic methods. Neoadjuvant chemotherapy can downstage tumors, allow for conservation surgery, spare ALND, and provide prognostic information. This study examined whether performing genomic assays on all HR+/HER2− breast cancers is feasible and if it affects treatment decisions. Patients with HR+/HER2− cancer identified on core biopsy underwent genomic assay testing and the information was made available to the care team. Of the patients included, 92% had grade 1–2, 93% had T1/2 tumors, and 86% were cN negative; patients with recurrent or metastatic disease were excluded.

The results were available for 81% of patients at their first medical oncology visit, for 34% at the time of their first surgical visit, and 70% and 38%, respectively, had their results mentioned in notes. Clinical and molecular risk was discordant in 38% of patients. The authors concluded that reflex genomic testing was technically feasible and potentially scalable, but not consistently documented in presurgical decision making.

### 13.3. Avoiding Axillary Staging in Elderly Women with Early-Stage Breast Cancer and the Impact on Survival

SNB is used to stage and guide treatment of early-stage breast cancer with less morbidity than ALND. SLNB is associated with pain in 33% of cases and paresthesia in 22% at 5 years. Choosing Wisely and the Society of Surgical Oncology (SSO) recommend avoiding SLNB in women aged >70 years with early-stage HR+/HER2− disease based on 2 RCTs that showed no difference in OS or recurrence between ALND and observation. However, surgeons continue to perform SLNB in these patients. Conflicting observational evidence has shown improved survival in patients who received axillary staging.

The objective of the study was to identify trends in axillary staging use, improve methodology from previous studies to potentially account for selection bias, and to incorporate HER2 status. Women aged ≥70 years with early-stage breast cancer in the SEER database from 2005 to 2015 were included, while women with metastatic/advanced disease, atypical histology, or neoadjuvant chemotherapy were excluded.

Axillary staging was performed in 84% of patients, with 22% nodal positivity. Increasing age was strongly associated with decreased axillary staging. Patients who did not receive staging had better disease characteristics (e.g., lower-grade tumors). When propensity score weighting was used to make the groups more similar, patients who did not receive staging were less likely to have chemo/radiotherapy, and had worse overall and breast-cancer-specific survival. However, breast-cancer-specific survival had a similar absolute risk.

A cohort of patients from Choosing Wisely showed similar results. Axillary staging was performed in 90% of patients. Increasing age was associated with decreased axillary staging. Once the data were adjusted for confounding factors (comorbidities, adjuvant therapy, and SLNB vs ALND), there was no difference in breast-cancer-specific survival.

The inferior survival outcomes in patients not undergoing axillary staging are probably the result of selection bias. It is possible that there is no impact on breast cancer survival and there might be a compensatory increase in axillary RT in the absence of axillary staging data.

## 14. Concluding Remarks

There have been significant improvements in all sub-groups of breast cancer, with systemic therapies of targeted agents, the streamlining of better surgical outcomes with oncoplasty and axillary clearance, RTs, AI technologies, imaging modalities, and survivorship. The patient voice was heard throughout each day, highlighting the importance of patient-reported outcomes. To end the symposium, breast cancer leaders who have made a significant contribution to Canadian breast cancer practice were honored in a short film montage featuring Drs. Karen Gelmon, Kathy Pritchard, Pamela Goodwin, Maureen Trudeau, André Robidoux, and Ivo Olivotto.

## Supplementary Materials

The following supporting information can be downloaded at: https://www.mdpi.com/article/10.3390/curroncol31040135/s1, Figure S1: Canadian Breast Cancer Symposium Program, 15–16 June 2023.

## Abbreviations

ACEi	angiotensin-converting enzyme inhibitor	ILD	interstitial lung disease
ADC	Antibody–drug conjugates	IMN	internal mammary node
AE	adverse event	IMNI	internal mammary node irradiation
AI	artificial intelligence	*JAMA*	*Journal of the American Medical Association*
AKT	RAC serine/threonine-protein kinase 1	Ki-67	kiel protein 67
ALND	axillary lymph node dissection	LICAP	lateral intercostal artery perforator
ALTJ	axillary lateral thoracic vessel junction	LYMPHA	lymphatic microsurgical preventing healing approach
ARB	angiotensin-receptor blockers	MIBC	multiple ipsilateral breast cancer
ASCO	American Society of Clinical Oncology	MRI	magnetic resonance imaging
BCS	breast-conserving surgery	NCCN	National Comprehensive Cancer Network
BCT	breast-conserving therapy	NIPT	non-invasive prenatal testing
BMI	body mass index	NLP	natural language processing
BRCA1/2	breast cancer gene 1 or 2	NSCLC	non-small cell lung cancer
cALND	complete axillary lymph node dissection	OS	overall survival
CBC	contralateral breast cancer	PALB2	partner and localizer of BRCA2
CBSC.	Canadian Breast Cancer Symposium	PARPi	poly (ADP-ribose) polymerase inhibitors
CDK4/6i	cyclin-dependent kinase 4/6 inhibitor	pCR	pathological complete response
CHEK2	checkpoint kinase 2	PD1	programmed cell death protein 1
CHUM	Centre Hospitalier de l’Université de Montréal	PD-L1	programmed cell death ligand 1
CiNA	Cancer Incidence in North America	PET	positron emission tomography
CMS	Centers for Medicare and Medicaid Services	PFS	progression free survival rate
CNS	central nervous system	Pi3K	phosphatidylinositol 3-kinase
CPS	clinical pathological scoring	PR	progesterone receptor
CT	computed tomography	PTEN	dual-specificity protein phosphatase (phosphatidylinositol 3,4,5-trisphosphate 3-phosphatase)
ctDNA	circulating tumor DNA	RCT	randomized controlled trial
CV	cardiovascular	RNI	regional nodal irradiation
DCIS	ductal carcinoma in situ	RT	radiotherapy
DFS	disease-free survival	RWD	real-world data
DHEA	dehydroepiandrosterone	RWE	real-world evidence
ECOG	Eastern Cooperative Oncology Group	SBRT	stereotactic beam radiotherapy
eFTS	enhanced first trimester screening	SEER	surveillance, epidemiology, and end results
ENE	extra-nodal extension	SG	sacituzumab govitecan
ESC	European Society of Cardiology	SLN	sentinel lymph node
ESR1	estrogen receptor 1	SOC	standard of care
FDA	Food and Drug Administration	SOGC	Society of Obstetricians and Gynecologists of Canada
GSM	genitourinary symptoms of menopause	SRS	stereotactic radiosurgery
HER2+/−	human epidermal growth factor receptor-2-negative/positive	SSO	Society of Surgical Oncology
HR	hazard ratio	TAD	targeted axillary dissection
HR+/−	hormone receptor-positive/negative	T-DM1	trastuzumab emtansine
HRD	homologous recombination deficiency	T-DXd	trastuzumab deruxtecan
IBR	immediate breast reconstruction	TKI	tyrosine kinase inhibitor
IC-OS	International Cardio-Oncology Society	TNBC	triple-negative breast cancer
IDC	invasive ductal carcinoma	US	ultrasound
IDFS	invasive disease-free survival	WBI	whole-breast irradiation
IHC	immunohistochemistry	WBRT	whole-brain radiotherapy
ILC	invasive lobular carcinoma		
