# Supplementary figures and images for: The Canadian Breast Cancer Symposium 2023 Meeting Report
Source: Curr Oncol. 2024 Mar 29;31(4):1774–802. doi: 10.3390/curroncol31040135 (PMC11049169; doi:10.3390/curroncol31040135)

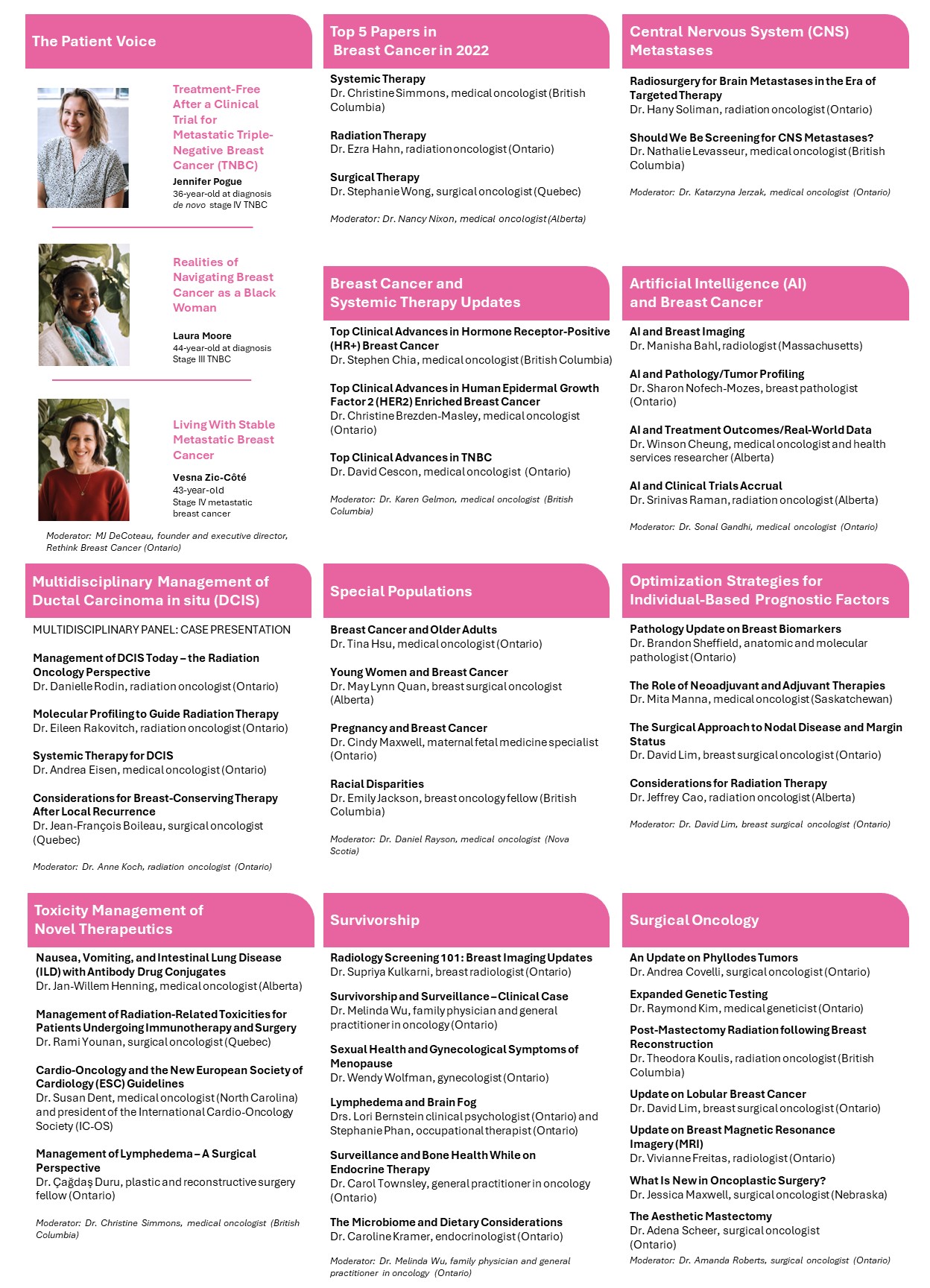

Supplement: Supplementary file 1 [file curroncol-31-00135-s001.zip › curroncol-2835048-supplementary.jpg]
